# Newly Generated and Non-Newly Generated “Immature” Neurons in the Mammalian Brain: A Possible Reservoir of Young Cells to Prevent Brain Aging and Disease?

**DOI:** 10.3390/jcm8050685

**Published:** 2019-05-15

**Authors:** Chiara La Rosa, Marco Ghibaudi, Luca Bonfanti

**Affiliations:** 1 Neuroscience Institute Cavalieri Ottolenghi (NICO), 10043 Orbassano, Italy; chiara.larosa@unito.it (C.L.R.); marco.ghibaudi@edu.unito.it (M.G.); 2 Department of Veterinary Sciences, University of Turin, 10095 Torino, Italy

**Keywords:** brain aging, brain structural plasticity, adult neurogenesis, brain reserve, cognitive reserve, doublecortin, immature neurons

## Abstract

Brain plasticity is important for translational purposes since most neurological disorders and brain aging problems remain substantially incurable. In the mammalian nervous system, neurons are mostly not renewed throughout life and cannot be replaced. In humans, the increasing life expectancy explains the increase in brain health problems, also producing heavy social and economic burden. An exception to the “static” brain is represented by stem cell niches leading to the production of new neurons. Such adult neurogenesis is dramatically reduced from fish to mammals, and in large-brained mammals with respect to rodents. Some examples of neurogenesis occurring outside the neurogenic niches have been reported, yet these new neurons actually do not integrate in the mature nervous tissue. Non-newly generated, “immature” neurons (nng-INs) are also present: Prenatally generated cells continuing to express molecules of immaturity (mostly shared with the newly born neurons). Of interest, nng-INs seem to show an inverse phylogenetic trend across mammals, being abundant in higher-order brain regions not served by neurogenesis and providing structural plasticity in rather stable areas. Both newly generated and nng-INs represent a potential reservoir of young cells (a “brain reserve”) that might be exploited for preventing the damage of aging and/or delay the onset/reduce the impact of neurological disorders.

## 1. Introduction

The aging of the brain, especially in the light of a progressive increase of life expectancy, will impact the majority of people during their lifetime, putting at stake their later life and that of their relatives. This cannot be seen only as a health problem for patients but as a more general, worrisome, social, and economic burden. In spite of fast and substantial advancements in neuroscience/neurology research, resolutive therapeutic solutions are lacking. Firstly, responsible for the current state of the art is the highly reduced capacity of the human brain for cell renewal during adulthood (most neurons are lifelong elements). For a long time, some hopes have been recognized in structural plasticity: The possibility for a “generally static” brain to undergo structural changes throughout life that may go beyond the modifications of synaptic contacts between pre-existing neuronal elements. During the last five decades, the discovery that the genesis of new neurons (adult neurogenesis) can still occur in some regions of the central nervous system (CNS) supported such hopes, suggesting that young, fresh neurons might replace the lost/damaged ones. Over the years, the field of brain plasticity has been continuously enriched by new findings and new possibilities/nuances of neural structural changes. Nevertheless, some pitfalls, difficulties, or even complex heterogeneities, also emerged. The real roles and functions of adult neurogenesis are far from being elucidated, and it appears clear that the new neurons can mainly serve physiological functions within the neural circuits, rather than being useful for repair. Interestingly, and adding further complexity, non-newly generated, immature neurons sharing the same molecular markers of the newly born cells are also present in the mature brain. While all these aspects of plasticity were revealed, some remarkable differences also started to emerge among mammalian species, indicating that, speaking of brain structural changes, mice and humans can be different due to evolutionary choices linked to different ecological niches and behavioral needs. On the other hand, data obtained in comparative medicine by studying animals in the wild strengthened the awareness that structural plasticity can be strongly modulated by lifestyle, allowing the brain to progressively change its structure and to maintain its “youth” throughout life, thus being the potential target of preventive strategies (in both aging and pathology).

This review article is intended to summarize and discuss the complex issue of structural plasticity heterogeneity in mammals on the basis of recent achievements (which, still, leave open many questions). Particular attention will be paid to the comparative aspect: The challenge of translating the results of neurobiological research to humans needs not only a thorough comprehension of the cellular and molecular mechanisms of brain structural plasticity, but also further knowledge of their evolutionary aspects and constraints.

### 1.1. Stability and Plasticity in the Nervous System

The CNS of mammals is a highly complex structure made up of a high number of neurons (10^11^), an even higher number of glial cells, and a huge number of synapses (estimated in 10^15^/mm^3^ in humans [1]). The system is further complicated by the extremely heterogeneous nature of its cellular elements and by the complexity of their intermingled processes (belonging to both neuronal and glial cells). On these bases, “a fundamental feature of CNS structure is its connectional, neurochemical and functional specificity, which allows specific cell types to be connected and to act in a relatively invariant way” [2]. Such architectural specificity is attained during development, then maintained and somehow fixed in the adult through a vast cohort of extracellular matrix molecules, adhesion molecules, and perineuronal nets [3,4,5,6,7]. During embryonic development, from neurulation to birth, massive structural changes allow morphogenetic processes (involving cell proliferation, cell migration, axonal/dendritic growth, synapse formation/elimination, programmed cell death), ultimately leading to brain and spinal cord formation and assembly of their neuronal circuits. Starting from the postnatal period, these processes are downregulated (with remarkable regional variations and following different temporal windows), providing a major stabilization of tissue architecture and neural connections, thus building up a prevalently non-renewable tissue [8,9,10]. This CNS “stabilization” largely accounts for the well-known incapability/scarce capability of mammals (depending on animal species, age, and brain region) to undergo neuronal regeneration and repair [8,11].

The postnatal downregulation of plastic processes does not lead to their complete disappearance everywhere in the CNS, leaving open possibilities for various types and degrees of structural changes (adult plasticity). In this context, plasticity, namely the ability to make adaptive changes related to the structure and function of the system [12,13,14], is an exception to the rule of a “static/stabilized brain” allowing the individuals to deal with environmental changes and challenges. Moreover, another important attribute of plasticity is that, in turn, it can be modulated by experience [15,16]. Hence, the good functioning of a mammalian brain (considering both its neural tissue and the individual behavior it can produce) implies a dynamic balance between stability and plasticity of its cellular components, leaving open possibilities for better adaptation to life in healthy individuals, and, maybe, for prevention of damage linked to aging, dementia, and neurological disorders. Though regionally specific, as to its temporal occurrence and persistence, CNS plasticity is usually maximal during early postnatal periods, in some cases allowing the continuous developmental growth and maturation of certain neuronal populations/brain areas, then progressively declining with age (see below).

During the last decades, a lot of research in developmental neurobiology tried to understand brain plasticity in mammals, in order to exploit the “range of structural changes” that are still possible in the relatively hard-wired adult CNS. If such intensive effort provided us with a huge amount of new information, it also started to reveal different levels of complexity: (i) Primarily, the different types of plasticity (often strictly intermingled/overlapping) and (ii) the evolutionary aspects that make the brains of mice and humans different [17,18,19,20]. As stated above, this review article is an attempt to update the current knowledge obtained in this complex and dynamic field, as well as trying to drive some suggestions for future perspectives in the prevention of age-related brain damage and cognitive decline, and/or in neuroprotection strategies for neurodegenerative diseases.

### 1.2. Different Types of Brain Structural Plasticity

**Figure 1:**
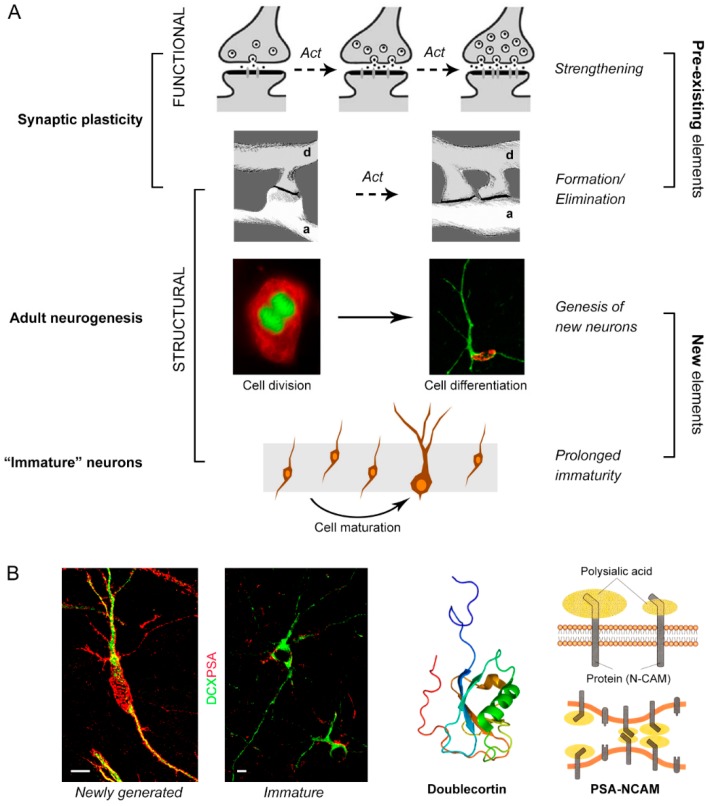
Different types of plasticity in the central nervous system. (**A**) Functional synaptic plasticity (strengthening of synaptic contacts) and structural synaptic plasticity (formation and elimination of synapses) involve modification of contacts among pre-existing elements (images inspired from [24,27]); *Act*, neuronal activity. Adult neurogenesis, a form of striking structural plasticity implying a change in the number of neurons, and prenatally generated neurons continuing to express molecules of immaturity for long time (“immature” neurons), are thought to provide a similar outcome through progressive maturation and, possibly, integration, thus providing examples of “whole cell structural plasticity”. (**B**) The main neuronal markers of immaturity (the cytoskeletal protein doublecortin, DCX, and the non-adhesive, polysialylated form of the neural cell adhesion molecule, PSA-NCAM) are co-expressed by both categories of neurons involved in whole cell structural plasticity (WCSP), namely, newly generated and non-newly generated immature neurons. Left, rabbit cerebellum; right, mouse cerebral cortex. Scale bars: 5 µm.

Considering its heterogeneity, plasticity is one of the most often used, but less defined terms in the neurosciences. Postnatal and adult CNS plasticity is what remains after the downregulation of developmental plasticity and can occur in different forms (Figure 1). First of all, neural plastic changes can be identified as “functional” and “structural”.

#### Functional and structural plasticity

Functional plasticity is associated with molecular changes that occur at the neuron/synaptic level, including modification in presynaptic transmitter release, postsynaptic receptor trafficking, signal transduction pathways, gene activation, and synthesis of new proteins, which ultimately can change the magnitude of synaptic transmission [21].

On a further level, the formation/elimination of synapses [22,23] can be considered as a form of structural plasticity, involving minimal modifications of the cell shape at the dendritic spine level [24,25]. These processes, by modifying the neuronal contacts, are crucial for learning and memory and can be exploited for compensatory responses in pathological conditions [26,27].

In the context of structural changes, we will consider here forms of extra-synaptic plasticity, in particular a “whole cell structural plasticity” (WCSP): When the entire cell is affected by the modification, thus changing the number, the entire morphology, and/or the identity of the cells, in addition to synaptic plasticity (which affects very small portions of the cell to modify the contacts among pre-existing neurons). Adult neurogenesis, consisting of a postnatal, stem cell-driven genesis of new neurons, which can ultimately integrate into the pre-existing neural circuits [28,29], is the typical example of WCSP.

Neuro-glial plasticity and gliogenesis, as modification of the neuronal-glial cell shape and relationships or glial cell number, respectively [8,30,31], can also fall in this category, along with the still controversial processes of in situ cell reprogramming [32,33]. Finally, a new emerging concept is that of “non-newly generated, immature neurons” [13,34,35], in which the potential for plasticity of the whole cell is evident (e.g., by the expression of cytoskeletal and membrane-bound molecules involved in overall structural changes: DCX and PSA-NCAM [6,36]), whereas the possible increase in number, due to final integration, is still debated (see below). Here, we will specifically address adult neurogenesis and immature neurons as forms of WCSP, which might represent a reservoir of young neurons, namely, a possible neuroanatomical substrate for brain reserve, in addition to synaptic plasticity.

### 1.3. Brain Structural Plasticity: Roles in Brain Repair and Preventive Strategies

Why is the study of brain structural plasticity considered so important? As stated above, the relative stability of the mammalian CNS accounts for its scarce potential for repair and regeneration: The reduced capacity for cell renewal during adulthood is strictly linked to the incapability to undergo efficacious reparative processes after damage [11,33,37,38,39]. For this reason, despite intensive research, most brain pathologies involving progressive degeneration or functional loss of neurons still fall into the category of incurable diseases. In modern societies, neurological diseases, dementia, and other age-related problems affecting the human brain represent a heavy health, social, and economic burden [40]. The progressive extension of human lifespan expectancy [41] will produce increasing numbers of individuals affected by neurological problems worldwide, thus making the issue of age-related dementia a global priority [42]. The translational importance of the studies on brain plasticity is obvious: Structural plastic changes are an exception to the limits of neural stability and may be the target for preventive and therapeutic approaches. Yet, paradoxically, in spite of a huge amount of literature published on neural plasticity (including all its forms and nuances), our brain seems to suffer a substantial lack of structural changes. Comparative studies carried out on non-mammalian vertebrates can provide an explanation: They clearly show that fish, amphibians, and reptiles do renew most neuronal populations throughout life and can perform regeneration after lesion [39,43]; by contrast, cell renewal and efficacious regenerative processes are no more a natural property of the mammalian nervous system, due to evolutionary constraints involving a combination of factors (e.g., brain complexity, scarcity of stem cells, incapability of cell de-differentiation and of re-activating developmental programs for regeneration, role of immune system; reviewed in [11,33,37,38]). As a result, apart from widespread synaptic plasticity, which operates only in very small portions of the pre-existing neuronal elements (dendritic spines, apical part of axons [44]), a WCSP involving changes in the overall shape and/or number of cells is a rare event in mammals, especially humans [19].

During the last three decades, most efforts in neurodevelopmental research have been directed to foster residual WCSP in the mammalian nervous system (mainly from stem cell-driven adult neurogenesis), with the aim of implementing endogenous neurogenic processes and/or reactivating intrinsic/silent programs of regeneration (reviewed in [38,45,46,47]). Different types of cellular reactivity can be experimentally induced after lesion in murine models. For instance, activation of stem/progenitor cells in the neurogenic niches [48,49] or local activation of neurogenic astrocytes [50,51] are elicited by inflammation/cell death and are followed by migration of neuroblasts toward the site of lesion. Nevertheless, these processes, though reminiscent of the first phases of tissue repair occurring in non-mammalian vertebrates [43,52], are ultimately abortive, since they do not lead to brain repair and cell replacement. They are mostly followed by disappearance of the newly born cell populations that frequently have a transient existence [49,51], during which they can exert “bystander” effects through the secretion of molecules/microvescicles in the site of lesion (reviewed in [38,53]), yet not leading to the replacement of damaged neurons and restoration of the tissue. In parallel to studies on endogenous adult neurogenesis, different types of stem/progenitor cells have been harvested, cultured, or produced in vitro (more recently including the induced pluripotent stem cells, which can be used as autologous cells) to be employed for transplantation. Yet, also in this case, no substantial efficacious treatments have been achieved in clinical trials for neurodegenerative diseases carried out in humans and based on stem cell products [54,55]. Out of 300 trials started before 2012 and using stem cells for neurological disorders in humans, not one led to efficacious treatment and final approval [56], which consists of high costs in terms of money and time. Even the most prominent scientists working on these issues and trying to use plasticity to foster brain repair agree that it would be premature to launch too many clinical trials and that further preclinical studies are needed [54,55]. On the whole, such efforts clash with the above-mentioned evolutionary constraints leading to substantial neural stability in mammals, not taking into account an aspect, which, in this research field, was underestimated since the beginning: The remarkable differences existing among mammalian species, with particular reference to murine models vs. humans. On the other hand, the same research conducted in the last few years and revealing a substantial failure in the direction of brain repair did highlight new, physiological roles of brain structural plasticity, which can be highly modulable by lifestyle and, thus, might be the target of preventive/protective strategies for aging and neurodegeneration. The next two paragraphs will be dedicated to elaborating on the opportunities and hitches of these complex issues.

### 1.4. Neurogenic Plasticity: The Dream of Brain Regeneration Facing Reality

**Figure 2:**
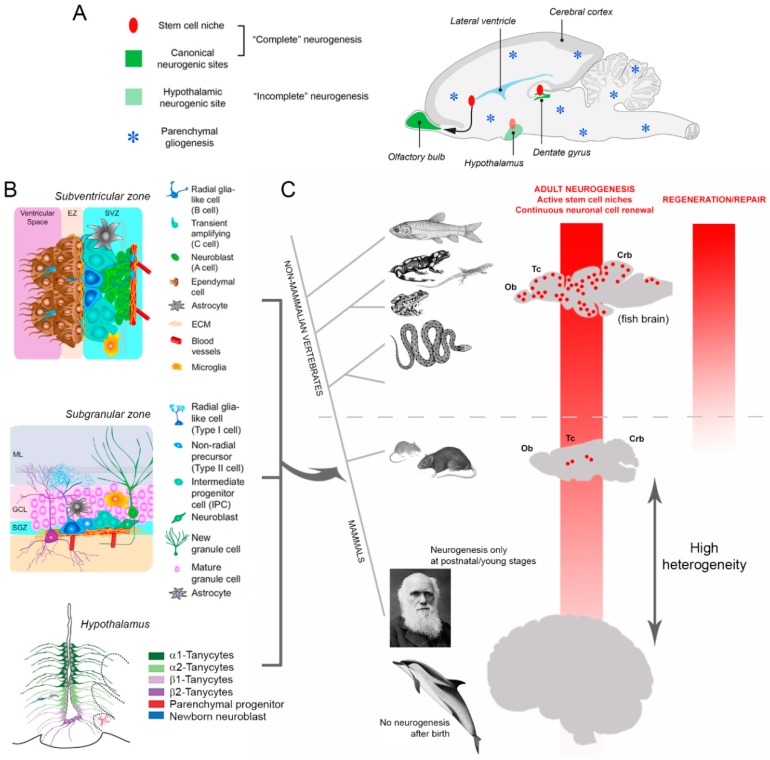
Adult neurogenesis in laboratory rodents and its reduction across vertebrates and mammals. (**A**,**B**) Remarkable knowledge has been gathered on the cellular and molecular mechanisms regulating the activity of the neural stem cell niches which provide new neurons throughout life in mice and rats. These neurogenic niches are restricted to three small brain regions (**A**, green), and only the subventricular zone (in the lateral ventricle wall) and the subgranular zone (in the hippocampus) are considered “canonical” neurogenic sites leading to “complete” neurogenesis. (**C**) Comparative studies revealed that the amount of stem cell-related plasticity strongly depends on evolutionary constraints: Adult neurogenesis and reparative capacity dramatically decrease from non-mammalian vertebrates to mammals, and, to a lesser extent, among mammals. Among the latter, the reduction follows a trend from small, lissencephalic to large, gyrencephalic brains, becoming restricted to early postnatal stages. Modified from [46] (with permission of Elsevier), [70] and [71].

After several evidences provided in mice and monkeys by Robert Altman in the 1960s [57] and in birds by Fernando Nottebohm in the 1980s [58], the genesis of new neurons in the adult mammalian brain was definitively proven in the 1990s by using viral vector techniques and thymidine analogues [59,60]. Two “canonical”, neurogenic sites are currently considered: The forebrain subventricular zone, lining the walls of the lateral ventricle (V-SVZ [29]), and the subgranular zone (SGZ), in the dentate gyrus of hippocampus [28] (Figure 2A). These neurogenic regions contain neural stem cells, which derive from radial glia (a glial—astrocytic—cell population originating during development), then remaining within the postnatal brain tissue as “niches”, namely, remnants of the embryonic germinal layers [61,62,63]. The neuronal precursors originating from the canonical neurogenic sites can differentiate into different types of neurons and functionally integrate into local network [64,65] displaying similarities and differences with respect to embryonic neurogenesis [66,67]. A third area of genesis, also linked to a periventricular germinal layer, has been identified in the hypothalamus [68,69] (Figure 2A); nevertheless, the final fate and functional integration of the hypothalamic newly born neuroblasts is still obscure, or at least not clear-cut as in the two canonical sites.

Since their discovery in the 1990s, adult neural stem cells, their niches, and their continuous renewal activity in the olfactory bulb and hippocampus were intensively studied in rodents, leading to deep knowledge of the niche cell components and molecular regulation (Figure 2B). Such research, fueled by the hope of using plasticity to replace lost/damaged neurons (both from endogenous—constitutive adult neurogenesis—and exogenous—cultured stem cells—sources), produced a huge amount of knowledge on the cellular and molecular mechanisms regulating/modulating the neural stem cell biology (now exceeding 10,000 scientific papers in a PubMed search for “adult neurogenesis”; reviewed in [29,65,72,73]).

Meanwhile, some neurogenic regions have been also described in addition to the neurogenic sites (previously referred to as “parenchymal” neurogenesis; reviewed in [10,74]). It is intended as a production of new neurons “external” to the restricted stem cell niches of the canonical neurogenic sites: Unlike these latter, it is extremely heterogeneous in terms of regional location, age, and physiological or pathological states, most importantly, depending on the animal species considered [10]. Examples of spontaneous neurogenesis have been found in lagomorphs. In rabbits, newly generated neurons are produced in two main regions of the adult CNS: The striatum (caudate nucleus, located in the forebrain) and the cerebellum. In the caudate is a population of newborn neuroblasts form longitudinally arranged, DCX-, and PSA-NCAM-immunoreactive chains of neuroblasts [75]. These neuroblasts are generated from clusters of proliferating cells that express the astroglial marker brain lipid-binding protein (BLBP), and about 1/6 of surviving cells differentiate into calretinin-positive striatal interneurons. Always in rabbits, a parenchymal genesis of Pax2+ interneurons resulting from further proliferation of cells of neuroepithelial origin has been described in the cerebellar cortex [76]. This process shows features of both delayed neurogenesis, extending until and around puberty [77], and persistent neurogenesis occurring, to a lesser extent, during adulthood [76]. Yet, the final outcome and role of all these neurons, which are spontaneously generated independently from remnants of germinal layers in the striatal and cerebellar tissue of rabbits, remain obscure.

The observations reported above, across the years contributed to generate the illusion that adult neurogenesis might be a rather widespread process, progressively tearing down the dogma of a static, non-renewable CNS. Nevertheless, this is far from true, for several reasons:

(i) If molecular and structural changes affecting the neural connectivity of pre-existing elements (synaptic plasticity, important in learning/memory and for compensatory effect after a lesion [22]) are rather widespread, the genesis of new neurons is restricted to the small neurogenic zones [10,74];

(ii) Only the newly born neurons produced in the two canonical neurogenic sites (SGZ and V-SVZ) do fully (and functionally) integrate into the neural circuits: This condition can be referred to as “complete” neurogenesis [10];

(iii) The ultimate fate of other (“parenchymal”) sites of cell proliferation remains unknown and no structural/functional outcome has been demonstrated until now (reviewed in [74]);

(iv) Most cells generated outside the neurogenic niches are glial cells [78,79];

(v) Even the complete neurogenesis occurring in the canonical neurogenic sites of mammals appears to be related to physiological needs and species-specific adaptations (see the eCollection in Frontiers [80]), see below;

(vi) Different locations of neurogenesis are strictly linked to the different animal species, behaviors, developmental stages, and ages. The latter point will be developed in paragraph 1.5.

It is more and more clear that adult neurogenic processes in mammals can subserve physiological functions (learning, memory, pattern separation, spatial navigation, adaptation to changing environment, sex-related aspects) linked to the genetics and ecological niches proper of the different animal species [81], not including a substantial role in brain repair. Even in the CNS of other vertebrates, the capacity for lesion-induced repair was likely a byproduct of evolution, namely, an epiphenomenon of the mechanisms underlying the development and maintenance (regeneration) of a particular structure [11,82] (Figure 2C). In addition, the association between adult neurogenesis and specific brain functions (an issue still far from being elucidated [17,81]) seems not to be an “a priori” established rule, since it can also vary: Bats, for instance, in spite of their spatial memory capacities linked to echolocation, display low levels of hippocampal neurogenesis [83]. A strong indication that adult neurogenesis could be strictly dependent on the physiological need for specific functions, which are usually very important for survival of the species, comes from our recent work on dolphins: In these aquatic mammals devoid of olfaction and brain olfactory structures, no periventricular neurogenesis was detectable after birth [84,85]. Odontocetes replaced olfaction with echolocation 40 million years ago, and now can live without neurogenesis (even in the hippocampus [86]), in spite of their high cognitive abilities.

### 1.5. Variations in Adult Neurogenesis among Mammals and its Overall Reduction in Large-Brained Species

Several studies carried out during the last two decades on mammalian species different from mice and rats started to show that adult neurogenesis occurrence, extension, rate, behavioral role(s), and function(s) can be quite heterogeneous among mammals (reviewed in [10,17,81,85,87]). This heterogeneity can occur at three main levels: (i) Different anatomical location, consisting of differences in the existence/location/outcome of neurogenic sites among species (linked to neuroanatomy and specific brain functions; see above the example of striatum and cerebellum in rabbits, and, outside of mammals, the seasonal neurogenesis in the bird telecephalon [58]; (ii) different rate among species, consisting of differences detectable in the same neurogenic region (e.g., the hippocampus of different species of bats or rodents [81,88]); (iii) different rate among ages within the same species (characterized by a general reduction after puberty and then further reduction with aging, but with remarkable interspecies differences linked to the different postnatal developmental/growth time scales [89,90,91]). In other words, the issue of adult neurogenesis reduction in mammals is made even more complex by the superposition of a “phylogenetic” decline (emerging when comparing different animal species) with a decline “linked to progressive ages” detectable in individuals of the same species (and usually present in all species).

No systematic, fully comparable studies (e.g., involving the count of dividing and DCX+ cells in different animal species, at corresponding ages, and in relation to the count of the different cell populations residing in the hippocampus, primarily granule cells) are available on a wide range of mammalian species to support this view or evaluate the importance of conservation in adult neurogenesis. An existent analysis, mostly based on rodent species, indicates that the rate of the neurogenesis varies widely, even between closely related members of the order Rodentia [88]. We know more about the “extremes”: High neurogenic rates in laboratory rodents (small-brained, lissencephalic) and vestigial/absent neurogenesis in humans and dolphins (large-brained, highly gyrencephalic). Differences are quite striking if considering the V-SVZ: Changes occurring in early postnatal human infants lead to substantial reduction in the activity of the stem cell niche (replaced in adults by an astrocyte ribbon and a hypocellular gap almost devoid of young neuroblasts) and to disappearance of the migratory stream connecting the periventricular niche with the olfactory bulb around 18 months of age [92]. Then, only rare migrating neurons are observed in the V-SVZ, and it remains unclear if these few cells can make the very long journey from the ventricle to their final destination in the olfactory bulb [92,93,94]. In mice, the V-SVZ retains actively dividing stem cell populations throughout life, thus providing continuous delivery of new neurons into the olfactory bulb (estimated in ~10,000 cells/day out of ~180,000 newly generated cells in the whole V-SVZ region in three-month-old mice [59,95,96]. A substantial reduction in the rate of neurogenesis occurs from young to adult age in both canonical neurogenic niches. Even in rodents, stem cell activity levels are quite reduced with age in the V-SVZ [97,98] and in the dentate gyrus (in C57BL/6 mice, the reduction in proliferation is 10-fold, from 0.76—percentage of Ki-67+ cells/granule cells—at two months to 0.08 at 9 months of age [99]). Also, the highly conserved neurogenesis occurring in the hippocampus reveals remarkable differences among mammals [81]. Striking examples are bats (very low levels of hippocampal neurogenesis—especially Microchiroptera—with high species-specific differences in its rate [83]) and dolphins (adult neurogenesis in the hippocampus of aquatic mammals is less investigated; a study conducted on different cetacean species failed to detect any DCX+ cells in the adult dentate gyrus [86]). In humans, a harsh debate has been fueled by recent reports driving different conclusions about the rate and temporal extension of hippocampal neurogenesis during adulthood [100,101]. Detailed studies carried out on postmortem and intraoperative samples of the human hippocampus from early gestation/postnatal stages to aging adults showed that proliferating progenitors and young neurons in the dentate gyrus sharply decline in early stages of life and only a few isolated young neurons were detected by 7–13 years of age [102,103]. These reports come to the conclusion that if neurogenesis continues in the adult dentate gyrus, this process must be extremely rare. In other recent studies claiming maintenance of neurogenesis in adult human hippocampus [104,105], various molecular markers were found associated to different stages of immature neurons not showing the typical aspect of recently-generated neuroblasts and rarely associated with cell proliferation. The Moreno-Jiménez work was particularly accurate in defining the best level/type of tissue fixation, thus increasing the possibility to detect DCX and PSA-NCAM specific staining in the postmortem human brain. Yet, surprisingly, in all these studies, no substantial cell proliferation was detectable, the different interpretations relying on the nature of the DCX+ and PSA-NCAM+ cells, defined as “immature” neurons [104,105]. When a longitudinal analysis carried out at different ages was performed, including early postnatal/young stages, cell division and neuroblast-like cells typical of the active stem cell niches were detectable [92,93,94,95,96,97,98,99,100,101,102,103].

On the whole, these data show that a dramatic drop in these phenomena actually occurs through ages in humans (especially concerning the existence and activity of the stem cell niches), whereas further studies are needed to establish more precisely the amount/type of immature neurons that persist through adulthood. Two main pitfalls remain to be solved in order to confirm/improve the current knowledge: (i) A lack of systematic, really comparable studies on the rate of neurogenesis carried out with the same method on different mammalian species (taking into account the complex variables involved, e.g., different ages, total number of mature neurons in each brain region, ecological/environmental/behavioral aspects); and (ii) the technical difficulties when dealing with brain tissues from unusual, large-sized species (e.g., different procedures/times of fixation, different post-mortem intervals; see [100,101,105]).

On the whole, an evident trend of reduction in adult neurogenesis has been shown to exist in mammals, from laboratory rodents to large-brained, long-living species. The link between low neurogenesis-long lifespan is broken up by the microchiroptera and naked mole-rats [106], both long-lived species but with small brains, thus suggesting that brain size might be the real feature linked to neurogenesis reduction [107,108].

## 2. Immature Neurons: A New Story in Brain Plasticity?

**Figure 3:**
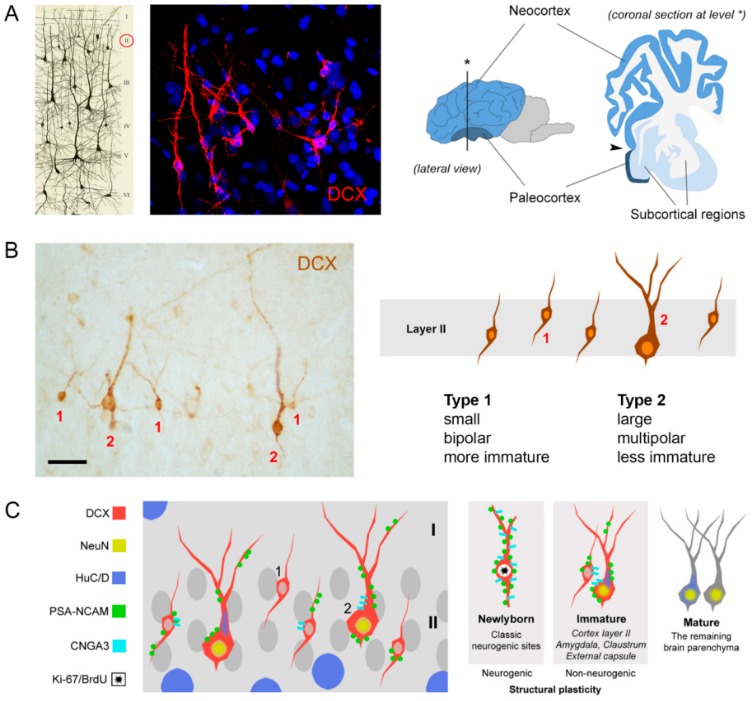
Non-newly generated immature neurons (nng-INs) in the cerebral cortex. (**A**) Cortical nng-INs are detectable by doublecortin (DCX) staining in layer II; in rodents, they are restricted to the paleocortex (right); arrowhead: Limit between paleo- and neo-cortex. (**B**) They occur in two different morphological types: Type 1 (small, bipolar) and type 2 cells (large, multipolar); (**C**) the nngIN cell types show different degree of immaturity: Type 1 cells are more immature than type 2 cells, as revealed by a different combination of markers. Modified from: (**A**) [71,108]; (**C**) [35].

Recent reports [13,34,109,110] led to the consideration of the possibility that another type of structural plasticity might exist: The occurrence of populations of “persistently immature” neurons that are not newly generated, yet remain in a relatively undifferentiated state in the adult by continuing to express molecular markers of immaturity, such as the polysialylated form of the neural cell adhesion molecule (PSA-NCAM [111,112]) and doublecortin (DCX [113]). These cells were firstly identified in layer II of the piriform and lateral enthorinal cortices of adult rats and mice [111,112] (Figure 3A), namely, the paleocortex or three-layered “allocortex”. They exist in two main morphological types: Small cells, with a 3–9 µm cell soma diameter (type 1 cells) and large cells, with 9–17 µm diameter (type 2 cells; the range depending on the animal species; Figure 3B). The small cells mostly show a simpler, bipolar morphology, whereas the large-sized cells frequently appear as pyramidal-like neurons (similar to the “principal cells” described by [34]; Figure 3B). The latter are usually far less abundant with respect to the former.

As to the origin of the immature neurons, the expression of molecular markers typically associated with adult neurogenesis has led many investigators of the past to consider them as newly generated (reviewed in [13]).

Yet, there is no evidence for cell division or cell migration, and more accurate studies employing 5-bromo-2-deoxyuridine (BrdU) injections followed by different survival times ultimately showed that the DCX+ cells of the cortical layer II are generated during embryogenesis and not in adulthood [34,35,109,114]. Hence, these neurons would persist in a prolonged immature state in the adult CNS. When this aspect can be experimentally proven (with local cell division markers, or better, with BrdU pulse labelling experiments followed by different survival times), the cells can be defined as “non-newly generated immature neurons” (nng-INs [35]), in order to avoid confusion with the newly born neuroblasts/neurons, which also remain immature for a short period of time (about three weeks in the canonical neurogenic sites of rodents [29,65]).

On the other hand, the fate of the cortical immature neurons is far less clear. It has been observed that the number of nng-INs in layer II of the rodent paleocortex declines as the animal age progresses [115,116,117], many of them becoming undetectable likely due to loss of expression of the immaturity markers. Similar results have been observed studying DCX expression in the cerebral cortex of guinea pigs [118], cats [119], and primates [120]. Recent data obtained by Rotheneichner et al. [121] by using a tamoxifen-inducible transgenic mouse model in which DCX-expressing cells can be permanently labeled with a green fluorescent protein (DCX-Cre-ERT2/Flox-EGFP), suggest that the nng-INs do not die with aging progression but mature as glutamatergic neurons. The maturation of these cells is supported by the increasing in dendritic ramification complexity and density of dendritic spine, and the appearance of the axon initial segments, necessary and sufficient to generate action potentials [121]. Yet, although these authors speak about a “structural integration”, a real, functional integration has not been shown to occur.

Since the fate of these cells is unclear, it becomes difficult to speculate on their function in the adult CNS. At present, the best hypothesis is that nng-INs of cortical layer II could constitute a pool of “reserve neurons”, which might differentiate under physiological or pathological circumstances to be recruited in cortical circuits after completing maturation [13,110,113,121]. As such, they might represent a form of WCSP, potentially available for different roles (new neurons slowly added through the years, new possible targets for synaptic contacts, young cells with paracrine functions); in the case they finally integrate, this could be considered a form of slow, “suspended” neurogenesis in which the cells are generated prenatally but their functional outcome starts during adulthood (see also [110]).

### Non-Newly Generated Immature Neurons: Young Cells for Large-Brained Mammals?

**Figure 4:**
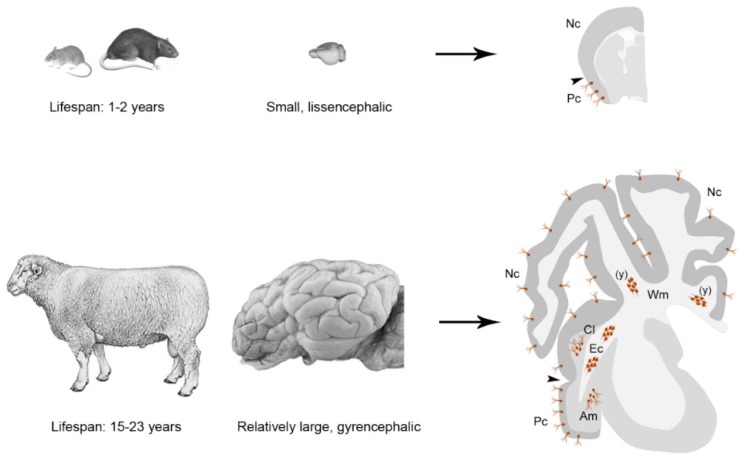
Cortical immature neurons (nng-INs, brown) are more abundant and widespread in large-brained, long-living mammals. Comparison between laboratory rodents (top) and sheep (bottom) showed a far more extended distribution of nng-INs in the latter, extending into the whole neocortex and in subcortical regions, such as amygdala and claustrum. Modified from [35,71].

Current data about nng-INs (and, more generally, immature neurons; see below) indicate that their distribution can substantially vary by considering different brain regions of different mammals. Whereas in mice and rats, most DCX+ neurons are confined to the ancient, three-layered paleocortex (allocortex), in rabbit, guinea pig, cat, some bat species, and some non-human primates, they were found also in neocortical areas, especially associative regions (reviewed in [13]). These results open the hypothesis that immature neurons might be more represented in animals endowed with relatively large and complex brains with respect to rodents, thus following a different trend if compared with adult neurogenesis [85,108]. In a recent study, by using markers of cell division (Ki-67) and BrdU pulse labelling in adult sheep [35], we revealed the presence of abundant DCX+ cell populations, born prenatally and not generated after birth (hence falling into the category of nng-INs), in different brain regions (Figure 4). Unlike previous reports, these cells were not restricted to the paleocortex (as in rodents) or to the cerebral cortex (as in other mammals studied so far), being also found in white and grey matter of pallial subcortical regions, such as the external capsule, the claustrum, and the amygdala [35]. Since sheep are endowed with a relatively large, gyrencephalic brain and a lifespan around 20 years, these results support the hypothesis that non-neurogenic plasticity might have been preserved better in long-living, large-brained mammals [108]. The extension of nng-INs to layer II of the whole neocortex (a brain region processing higher cognitive functions), and to neuroanatomical domains involved in the coupling of emotions and conscious perception (amygdala and claustrum [122,123]), does support the idea that nng-INs could have higher importance in highly complex brains and rather stable brain regions. The neocortex, namely, the place for conceptual processing in humans, is not endowed with neurogenic plasticity such as the olfactory bulb and the hippocampus. This should not be seen as a loss, rather as a need for more stability necessary for storing long-term memories or plans for the future. Hence, “there may be an advantage in keeping your old neurons without adding new ones, when the aim is to acquire and preserve complex knowledge during many decades of life” [124]. It has been hypothesized that layer II, in addition to connections with deeper layers, mainly contains associative neurons responsible for connecting different regions of the cerebral cortex, rather than projection neurons [125]. This aspect fits well with their increasing occurrence in brains characterized by high expansion of the neocortex, which are known to be endowed with more extended “associative” areas [126,127]. With increasing size of the brain and extension of the cerebral cortex, association areas take up an increasingly larger percentage of the cortex and are essential for mental functions that are more complex than detecting basic dimensions of sensory stimulation, for which primary sensory areas appear to be necessary [128]. In that sense, nng-IN could be the highest form of WCSP acquired by the mammalian brain.

In conclusion, we still do not know if nng-INs, in terms of plasticity, might represent an evolutionary choice of large-brained, gyrencephalic mammals and for a brain region that has evolutionarily expanded to become mostly responsible for higher cognitive functions (the neocortex [126]). Although current data strongly support this view, further studies spanning through widely different mammalian species and orders, including humans, are needed.

## 3. The Concept of “Cognitive Reserve” and the Missing Substrate(s)

### 3.1. Brain Reserve and Cognitive Reserve

In neurology, the largely debated concept of *reserve* is employed to explain discrepancy between the individual level of brain pathology and the expected cognitive performance. It is particularly important for aging, dementia, and all forms of brain damage. Different definitions have been used, such as brain reserve, cognitive reserve, and brain maintenance [129,130]. In *brain reserve*, the effective measures of reserve are anatomical, such as brain volume, head circumference, synaptic count, and dendritic branching. In this case, when a certain amount of the brain is compromised by aging or pathologies, the functional impairment is inevitable. Since the brain is able to be more plastic than once imagined, in the last few years, brain reserve as a “passive model” has been discussed [130], introducing the concept of *cognitive reserve*: The brain tries to face up to the degeneration and damage using pre-existing cognitive processes or recruiting compensatory processes. Responsible for cognitive reserve are innate intelligence and different lifetime experiences, such as educational and occupational attainment and leisure activity [131].

All these variables, taken together, could allow some people to set skills or repertoires to face up to aging and diseases better than others. For example, it is largely described that lower educational and occupational attainment is associated with a higher risk of dementia and Alzheimer’s disease incidence [132]. A study carried out on Lebanese older adults showed that non-dementia people received more education, attained higher occupation, and were physically more active than participants of the study diagnosed with dementia [133]. In the Nun Study (involving 93 sisters), it is largely demonstrated that education reflects the wideness of cognitive ability and neurological development in early life and the cognitive reserve in late life: In this case, the linguistic ability in early life (using handwritten autobiographies from youth) and the Alzheimer’s disease in late life (using post-mortem brains) have been evaluated. Lower linguistic ability in early life are associated with low cognitive test scores in late life; moreover, among the dead sisters, Alzheimer’s disease was present in all the nuns with lower linguistic ability and in none of those with higher abilities [134]. Finally, Stern and colleagues, using fMRI, identified a “task-invariant” cognitive reserve network that is active during the performance of many disparate tasks, whose expression correlates with a measure of IQ [135].

All these data strongly indicate that protective/compensatory mechanisms could be responsible for resistance to neurological diseases/dementia or significant delay of the clinical symptoms. Despite fragmentary information due to obvious difficulties encountered in studying humans, it is clearly emerging that some individuals with Alzheimer disease-like neuropathology do not become symptomatic during their lifetime, and that it may require more pathology for the individuals with greater brain reserve to manifest clinical dementia [136,137,138,139]. Individuals that remain cognitively intact in spite of having accumulation of amyloid plaques and neurofibrillary tangles to an extent comparable to that normally observed in fully symptomatic Alzheimer’s disease (AD) are referred to as “non-demented with Alzheimer’s neuropathology” [138,140]. They have also been termed “cognitively successful aging”, “asymptomatic AD”, “resilient AD”, and “preclinical AD” (reviewed in [138]). Yet, very little is known about the cellular mechanisms and biological processes occurring within the nervous tissue and actually involved in producing the protective effects.

### 3.2. Which are the Neuroanatomical Substrates of Brain/Cognitive Reserve?

First of all, the “reserve” should not be figured out as a single underlying process, resource, or entity, rather as a set of different processes acting at different temporal and spatial scales [141]. Then, though brain and cognitive reserve are two different concepts (“brain reserve might be considered the hardware while cognitive reserve the software” [130]), they are largely connected: Different kinds of experience have been associated with structural changes in the brain. It is well known that enriched environment increases size and weight of the brain, stimulates hippocampal neurogenesis, and ameliorates chronic disease phenotypes [16,73]. Reserve could be an improvement of neural resources, accumulated before the aging-related processes or the onset of age-related diseases, reducing the effects of neural decline [142]. In turn, some people are able to maintain their brain and their brain reserve more than others, probably also thanks to their cognitive reserve (brain maintenance [130]). This could be due to the structural changes induced in the brain (modification of the hardware) as a consequence of individual lifestyles aimed at improving the cognitive reserve. Nevertheless, almost nothing is known concerning which structural plastic processes actually underlie the establishment of a reserve. Technical/methodological limits still prevent a full reconstruction of the events occurring in the whole brain during the lifespan of an individual. Most information deals with the two extremes of the entire process: From the cellular/molecular studies carried out on specific systems in animal models (often focused on a single neuronal population or a small portion of the cells, e.g., dendritic spines) to the longitudinal studies carried out in humans that highlight the effects on the cognitive performances (with little resolution regarding the structural changes at a cellular level). In the middle, there are only histopathological specimens on postmortem brains and fMRI studies dealing with entire brain areas with limited resolution, but not giving substantial information to the progressive processes of structural changes occurring at a cellular level in the whole brain through lifespan.

For a long time, synapse number and brain size have been considered the hypothetical neuroanatomical substrates for brain reserve, generally figuring out an overall change in neural connections [143,144]. The limitation of the range of changes occurring in brain reserve to modifications occurring between pre-existing neural elements is an interpretation linked to the classic view of the brain “stabilized” under the profile of its cellular components. Even after the discovery of adult neurogenesis, the fact that new neurons can be added only in the two canonical neurogenic zones but not in the cerebral cortex (it is admitted that only a small number of interneurons can be produced in the cortex of rodents [145]), would leave this part of the brain, mostly involved in dementia, with the only hope of synaptic changes. Research carried out during the last three decades has added other possibilities (summarized above), some of which linked to WCSP. It is well known that different lifetime experiences can shape brain structure in animal models by creating new neurons in the hippocampus [65]. The enriched environment, largely investigated in rodents, is one of the best examples to show how experiences can induce plastic changes in the brains at different levels, by stimulating different processes such as neurogenesis, synaptogenesis, gliogenesis, angiogenesis, cortical thickness, white matter integrity, dendritic length and density, and modulation of neurotransmitter and neurotrophin expression [15,16,73,143,144]. By shifting to humans, all these changes may offer a reserve contributing to allow different people to face up to cognitive decline linked to aging, dementia, and brain injury. These conditions lead to cell loss, changes in the anatomy of neurons, with loss of synapses and dendritic spines, and dendritic tree degeneration [146]. Yet, moving from animal models to the human brain, the data are fragmentary and incomplete. Several experiences seem to influence brain structures and network in physiological and pathological conditions and magnetic resonance structural neuroimaging provides a tool to evaluate changes in terms of increase of gray or white matter volumes. Bilingualism, for example, exerts neuroprotective effects against neurodegeneration: Increased gray matter density has been reported in the anterior cingulate cortex, in the left inferior parietal cortex, in the left caudate, and in the left putamen, regions of the basal ganglia, all areas involved in second-language acquisition and proficiency [147].

**Figure 5:**
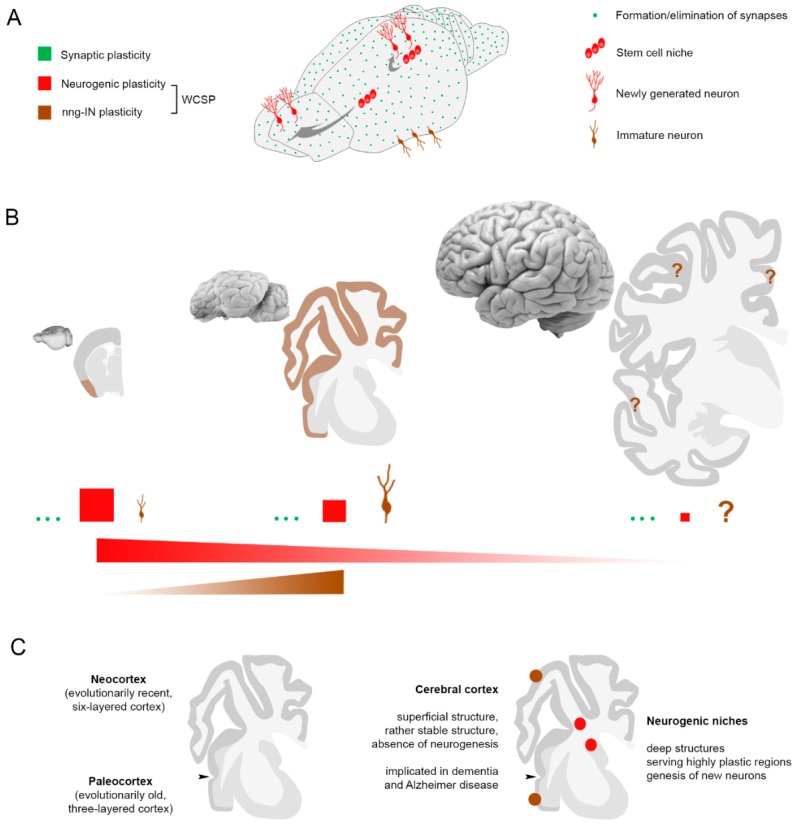
Knowledge on brain structural plasticity, including their relative occurrence/importance in different mammals, is rapidly evolving. (**A**) In addition to widespread forms of plasticity involving the contacts among pre-existent neuronal elements (synaptic plasticity), examples of “whole cell structural plasticity” (WCSP) are also present in certain brain regions. Active neurogenic niches produce new neurons in the hippocampus and lateral ventricle wall, whereas non-newly generated immature neurons (nng-INs) can be found in layer II of the paleocortex. (**B**) Though synaptic plasticity can be considered to be universally present, both forms of WCSP appear to be more species-specific: Adult neurogenesis decreases from mice to man, whereas nng-INs seem to increase from the small brain of rodents to the larger brain of sheep (see also Figure 4). (**C**) Unlike neurogenic plasticity (red), deeply located within the hemispheres and serving highly plastic brain regions (dentate gyrus and olfactory bulb), nng-INs (brown) are present in the relatively stable cerebral cortex (endowed with synaptic plasticity but not neurogenesis), responsible for the higher cognitive functions and frequently affected by dementia.

As to the possible role of WCSP in humans, we now know that adult neurogenesis is mostly inactive in adult individuals (discussed above). The genesis of new neurons might be exploited and stimulated during juvenile ages, which could be beneficial in progressively building up and finely sculpting the hippocampal circuitries [148]. Although this would leave uncovered the cerebral cortex (in which substantial addition/renewal of neurons is lacking, even in rodents), the recent evidence for prenatally generated, cortical immature neuronal populations (nng-INs) might open the possibility for a reserve also in this brain region, with special reference to large-brained mammalian species (Figure 5).

## 4. Immature Neurons: A Reservoir of Young Cells for the Adult/Aging Brain?

Independently from any specific physiological function (at present unknown), the novel population of “immature” neurons (nng-INs) raise interest in the general context of mammalian structural plasticity, potentially representing an endogenous reserve of “young”, plastic cells present in cortical and subcortical brain regions. Finding more about such cells, especially regarding their topographical and phylogenetic distribution, their fate with increasing age, and the external/internal stimuli that might modulate them, would open new roads for preventive and/or therapeutic approaches against age-related brain damage and cognitive decline. In addition to the nng-INs, it is worthwhile to note that even in reports supporting the existence of adult human hippocampal neurogenesis, the authors frequently refer to the DCX+ and PSA-NCAM+ cells as “immature neurons”, and only a few proliferating cells were found in adult individuals [104,105,149]. This observation acquires further interest in the light of previous descriptions of newly born DCX+ neurons, which can persist in the hippocampus of sheep and monkeys long after their proliferation, in a sort of prolonged maturation (from 3 to 6 months [150,151,152]). Taken together, these data suggest that maturation of neurons in the neurogenic sites of mammalian species living longer than rodents might be delayed in parallel with a reduction in the rate of neurogenesis. A current hypothesis is that in the large-brained, long-living humans the neurons generated at young ages might mature slowly, maintaining plasticity and immaturity for very long periods.

Hence, immature neurons, intended as both newly generated (in neurogenic sites) and non-newly generated (nng-INs in cortex and subcortical regions), might represent a form of WCSP granting a “reserve” of young neurons in the absence of continuous cell division. In this context, solid evidence suggests that “adult” neurogenesis in mammals should not be considered as a constitutive, continuous process taking place at the same rate throughout life, but rather as an extension of embryonic neurogenesis, which can persist for different postnatal periods by decreasing (even ceasing) at different ages and in different brain regions [10,20,148]. There is no sharp boundary between developmental processes and subsequent tissue maintenance and aging processes and some events, such as adult neurogenesis, have all the hallmarks of late developmental processes [148]. In that sense, adult neurogenesis is not at all similar to the cell renewal/regenerative processes known to occur in other stem cell systems, such as the skin, blood, or bone [153,154]; rather, it is characterized by progressive neural stem cell/progenitor depletion [155], the cell addition being directed at the completion of organ or tissue formation, not at the replacement of lost cells [148]. This aspect is more prominent and precocious in large-brained mammals, especially humans [85,107,124].

For the above-mentioned reasons, it appears that the complex mammalian brain, in the play between plasticity and stability, has chosen the possibility to delay some developmental/growth processes during the postnatal and adult periods (with rates and temporal windows/critical periods depending on the animal species), thus allowing the appropriated assembly/growth/maturation of specific brain regions and neuronal populations through life experiences. Such slow, progressive maturation of specific neuronal populations, especially in the hippocampus and cerebral cortex, might be, among others, a further substrate of the brain/cognitive reserve (involving WCSP), thus representing the target of lifestyle strategies (in the future, after having unraveled possible modulation mechanisms, also pharmacological treatments) for preventing/reducing the impact of damage linked to brain aging and/or delay the onset or reduce the impact of neurological disorders. For instance, it has been demonstrated (in murine models) that some substances (e.g., Fluoxetine) are able to increase hippocampal neurogenesis and possibly reopen critical period plasticity during adulthood [156,157] (Guirado and Castrén, 2018; Micheli et al., 2018). These treatments could act on a small reserve of immature neurons even in old individuals. In spite of the reduction of a “reserve” with age (both neurogenesis and immature neurons progressively drop with age), many observations suggest that the addition of small numbers of new neurons in the mature neural circuits can be relevant to their functioning (see [158,159]). At the extreme, it has been shown that even single neuronal cells can be functionally significant and have behavioral consequences [160,161]. Finally, the immature neurons (here intended more generally as “young neuronal cells”) are known to exert a “bystander” paracrine effect in both physiological and pathological states [38,53], thus representing a potential target for neuroprotective treatments and for the prevention of aging rather than cell replacement. In this direction, one future perspective should be a search for possible environmental stimuli that might modulate these cells (e.g., modify their maturity/immaturity state, foster/inhibit their integration, modify their paracrine activity) in order to either maintain the “reserve of young neurons” or promote their use within the pre-existing neural circuits.
